# GASTRIC CANCER WITH POSITIVE EXPRESSION OF ESTROGEN RECEPTOR ALPHA: A CASE SERIES FROM A SINGLE WESTERN CENTER

**DOI:** 10.1590/0102-672020210002e1635

**Published:** 2022-01-31

**Authors:** Alice Cristina Castro DA SILVA, Marina Alessandra PEREIRA, Marcus Fernando Kodama Pertille RAMOS, Leonardo CARDILI, Ulysses RIBEIRO, Bruno ZILBERSTEIN, Evandro Sobroza de MELLO, Tiago Biachi de CASTRIA

**Affiliations:** 1 Centro Universitário do Planalto Central Apparecido dos Santos - UNICEPLAC, - Brasilia - Distrito Federal - Brasil; 2 Instituto do Câncer, Hospital de Clinicas (HCFMUSP), Faculdade de Medicina, Universidade de São Paulo - SP - Brasil.

**Keywords:** Stomach Neoplasms, Estrogen Receptor alpha, Immunohistochemistry, Molecular Targeted Therapy, Prognosis, Neoplasias Gástricas, Receptor alfa de Estrogênio, Imuno-Histoquímica, Terapia de Alvo Molecular, Prognóstico

## Abstract

Despite advances in therapies, the prognosis of patients with advanced gastric cancer (GC) remains poor. Several studies have demonstrated the expression of estrogen receptor alpha (ERa); however, its significance in GC remains controversial.

**AIM:**

The present study aims to report a case series of GC with ERa-positive expression and describe their clinicopathological characteristics and prognosis.

**METHODS:**

We retrospectively evaluated patients with GC who underwent gastrectomy with curative intent between 2009 and 2019. ERa expression was assessed by immunohistochemistry through tissue microarray construction. Patients with ERa-negative gastric adenocarcinoma served as a comparison group.

**RESULTS:**

During the selected period, 6 (1.8%) ERa-positive GC were identified among the 345 GC patients analyzed. All ERa-positive patients were men, aged 34-78 years, and had Lauren diffuse GC and pN+ status. Compared with ERa-negative patients, ERa-positive patients had larger tumor size (p=0.031), total gastrectomy (p=0.012), diffuse/mixed Lauren type (p=0.012), presence of perineural invasion (p=0.030), and lymph node metastasis (p=0.215). The final stage was IIA in one case, IIIA in three cases, and IIIB in two cases. Among the six ERa-positive patients, three had disease recurrence (peritoneal) and died. There was no significant difference in survival between ERa-positive and ERa-negative groups.

**CONCLUSIONS:**

ERa expression is less common in GC, is associated with diffuse histology and presence of lymph node metastasis, and may be a marker related to tumor progression and worse prognosis. Also, a high rate of peritoneal recurrence was observed in ERa-positive patients.

## RESUMO - RACIONAL

Apesar do avanço nas terapias, o prognóstico de pacientes com câncer gástrico (CG) avançado permanece ruim. Vários estudos demonstraram a expressão do receptor de estrogênio alfa (REa), porém seu significado no CG permanece controverso.

**OBJETIVO:**

relatar uma série de casos de CG com expressão de REa-positivo, e descrever suas características clínicopatológicas e prognóstico.

**MÉTODOS:**

Avaliamos retrospectivamente os pacientes com CG submetidos à gastrectomia com intenção curativa entre 2009 e 2019. A expressão do REa foi avaliada por imuno-histoquímica por meio da construção de microarranjos de tecido (TMA). Pacientes com adenocarcinoma gástrico ERa-negativos serviram como grupo comparação.

**RESULTADOS:**

No período selecionado, foram identificados 6 (1,8%) CG REa-positivos entre os 345 CG analisados. Todos os ERa-positivos eram homens, com idades entre 34-78 anos, tinham CG do tipo difuso de Lauren e pN+. Comparado aos REa-negativos, os CG REa-positivos associaram-se a maior diâmetro (p=0,031), gastrectomia total (p=0,012), tipo de Lauren difuso/misto (p=0,012), presença de invasão perineural (p=0,030) e metástase linfonodal (p=0,215). O estágio final foi o IIA em um caso; IIIA em três e IIIB em dois casos. Entre os 6 pacientes REa -positivos, 3 tiveram recorrência da doença (peritoneal) e morreram. Não houve diferença significativa na sobrevida entre os grupos REa-positivo e negativo.

**CONCLUSÃO:**

A expressão do REa é menos comum no CG, estando associada à histologia difusa e presença de metástases linfonodal, podendo servir como um marcador relacionado à progressão tumoral e pior prognóstico. Além disso, uma alta taxa de recorrência peritoneal foi observada em pacientes ERa-positivos.

## INTRODUCTION

Gastric cancer (GC) is the fourth most common type of cancer worldwide, ranking third in cancer mortality^6^. It is diagnosed more frequently in advanced stages and, despite advances in therapies in recent years, the effectiveness of therapeutic options has still been low - both in locoregional and metastatic cancer^11^ ^,^ ^20^ ^,^ ^21^.

The use of a monoclonal antibody that interferes with the activation of human epidermal growth factor 2 (HER2) was the first step toward the target molecular therapy of GC. Trastuzumab has shown benefit in the survival of patients with metastatic GC^3^. However, since the approval of trastuzumab, several studies have been conducted in investigating other target agents^7^ ^,^ ^14^.

Estrogen is part of a class of steroids involved not only in the regulation of the reproductive system but also in the cardiovascular, neuroendocrine, and musculoskeletal systems. There are two subtypes of estrogen receptors (ERs): alpha (a) and beta (ß), which have variable tissue distributions and different biological functions^2^ ^,^ ^5^ ^,^ ^16^ ^,^ ^24^.

The blockade of REa has the capacity to suppress the malignant behavior of GC cells in vitro through the modulation of the expression of p27, p21, p53, cyclin D1, and E-cadherin^23^. However, some controversies regarding the expression of ERa in GC and its prognostic impact in these patients still remain^8^ ^,^ ^16^.

Accordingly, although hormone therapy has been used for decades in tumors with positivity for hormone receptors, such as breast and prostate cancer, in GC, more studies are still needed to determine their clinicopathological and prognostic significance^8^. Thus, the present study aims to report a case series of GC with ERa-positive expression and describe their clinicopathological characteristics and prognosis. Also, their characteristics and survival outcomes were compared with ERa-negative GC.

## METHODS

All GC patients, who underwent gastrectomy with curative intent, between 2009 and 2019, were retrospectively evaluated from our medical database. Inclusion criteria were as follows: histological confirmation of gastric adenocarcinoma and formalin-fixed, paraffin-embedded (FFPE) blocks of tissue available for analysis. Exclusion criteria were as follows: palliative resections, emergency surgeries, and systemic metastatic disease (M1).Total or subtotal gastrectomy and lymph node dissection were performed based on the guidelines of the Japanese Gastric Cancer Association^11^ and in accordance with the guidelines of the Brazilian consensus^4^. The pathological tumor stage was defined according to the 8th edition of TNM, as proposed by the International Union Against Cancer (UICC)^1^.

Clinical, surgical, and pathological variables, including sex, age (years), body mass index (BMI) (kg/m^2^), American Society of Anesthesiologists classification (I/II or III/IV), hemoglobin (g/dL), albumin (g/dL), the extent of resection, the extension of lymphadenectomy, tumor size (cm), histological Lauren type, lymphatic invasion, venous invasion, perineural invasion, number of lymph nodes, and pTNM stage, were evaluated.

Since this is a noninterventional and retrospective study, informed consent was not required from each patient. The study was approved by the Ethical Committee and Institutional Review Board (plataformabrasil.saude.gov.br; registration number CAAE: 38156720.0.0000.0068).

### Tissue microarray construction and Immunohistochemistry

All hematoxylin and eosin (H&E)-stained slides were reviewed, and representative tissue samples were selected for each case. Three cores of tumor tissue and two cores of adjacent mucosa were punched out from FFPE blocks and arrayed in a new tissue microarray (TMA) block using a precision mechanized system. Sections (4-μm thick) from each TMA block were performed for H&E and immunohistochemical staining.

Immunohistochemistry (IHC) was performed using a Ventana BenchMark ULTRA automated staining system with a primary monoclonal antibody for ERa (Clone SP1; Ventana Medical Systems, Inc.; reference number: 790-4324), according to the manufacturer’s instructions.

Cases were evaluated based on brown cytoplasmic and/or nuclear staining, and the staining intensity was graded by the Allred score system (range 0-8)^2^, expressed as the sum of scores representing the proportion and staining intensity of negative and positive tumor cell nuclei. Cases with score 2 were designated as positive for ERa expression. The immunoreactivity was viewed by two pathologists independently in a blinded manner. If there was a difference between the two observers, these slides were reanalyzed by both investigators using a multiheaded microscope.

### Statistical analysis

Descriptive statistics included frequencies with percentage for nominal variables and mean with ±standard deviation (SD) for continuous variables. Fisher’s exact test analysis was used for categorical data and t-test for continuous data. Survival was estimated using the Kaplan-Meier method, and differences in survival curves were examined using the log-rank test. Disease-free survival (DFS) was calculated from the date of surgery to the date of recurrence or the last follow-up. Overall survival (OS) was defined as the time between surgery and death of any cause or last follow-up. All data were analyzed using SPSS version 20.0 (SPSS Inc., Chicago, IL). Statistical significance was defined as p<0.05.

## RESULTS

During the selected period, a total of 345 patients were included in the study and evaluated for ERa expression. The majority of GC patients were men (60%), with a mean age of 62.4 years. Subtotal gastrectomy was the most performed type of surgery and 83.7% of patients underwent D2 lymphadenectomy.

**Figure 1 -:**
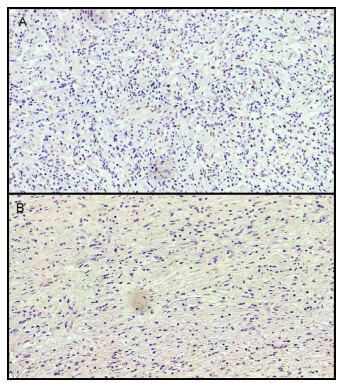
Immunohistochemical findings: (A) gastric adenocarcinoma positive for ERa and (B) adenocarcinoma negative for ERa staining (20×).

According to the ER evaluation, 6 (1.8%) patients were identified as ERa-positive. The remaining 339 (98.2%) patients with ERa-negative served as a comparison group (Figure 1).

Table 1 shows the characteristics of the two ERa groups. Total gastrectomy (p=0.012), larger tumor size (p=0.031), diffuse/mixed Lauren type (p=0.012), presence of perineural invasion (p=0.030), and lymph node metastasis (p=0.215) were associated with ERa-positive group. There were no statistical differences regarding gender, age, number of lymph nodes dissected, and TNM between the groups.

**Table 1 -:** Clinical and pathological characteristics of patients with gastric cancer according to the expression of ERa.

Variables	ERa-negative	ERa-positive	p
	n=339 (%)	n=6 (%)	
Sex			
Women	138 (40.7)	0 (0)	0.085
Men	201 (59.3)	6 (100)	
Age (years)			
Mean (SD)	62.4 (11.7)	62.4 (15.0)	0.999
Body mass index (kg/m²)			
Mean (SD)	24.1 (5.5)	21.9 (1.7)	0.347
ASA classification			
I/II	293 (86.4)	6 (100)	0.605
III/IV	46 (13.6)	0 (0)	
Hemoglobin (g/dL)			
Mean (SD)	12.1 (2.3)	13.9 (2.0)	0.060
Albumin (g/dL)			
Mean (SD)	4.1 (1.8)	4.2 (0.5)	0.888
Type of resection			
Subtotal	178 (52.5)	0 (0)	**0.012**
Total	161 (47.5)	6 (100)	
Extent of lymphadenectomy			
D1	55 (16.2)	1 (16.7)	1.0
D2	284 (83.8)	5 (83.3)	
Tumor size (cm)			
Mean (SD)	5.0 (3.2)	7.8 (3.2)	**0.031**
Histological type			
Intestinal	179 (52.8)	0 (0)	**0.012**
Diffuse/mixed	160 (47.2)	6 (100)	
Lymphatic invasion			
Absent	170 (50.1)	1 (16.7)	0.215
Present	169 (49.9)	5 (83.3)	
Venous invasion			
Absent	231 (68.1)	5 (83.3)	0.669
Present	108 (31.9)	1 (16.7)	
Perineural invasion			
Absent	170 (50.1)	0 (0)	**0.030**
Present	169 (49.9)	6 (100)	
No. of lymph nodes retrieved			
Mean (SD)	39.2 (18.6)	36.7 (14.3)	0.744
pT			
T1/T2	127 (37.5)	1 (16.7)	0.418
T3/T4	212 (62.5)	5 (83.3)	
pN			
N+	149 (44)	0 (0)	**0.039**
N1	190 (56)	6 (100)	
pTNM			
I/II	185 (54.6)	1 (16.7)	0.099
III/IV	154 (45.4)	5 (83.3)	

SD, standard deviation; ASA, American Society of Anesthesiologists; BMI, body mass index. *P* values in bold are statistically significant.

Table 2 summarizes the characteristics of each six ERa-positive patients. All ERa-positive patients were men who aged 34-78 years. Also, all cases had Lauren diffuse GC and pN+ status. The final stage was IIA in one case, IIIA in three cases, and IIIB in two cases. All ERa-positive patients received some chemotherapy (CMT) regimen: (1) neoadjuvant CMT, (2) adjuvant CMT, or (3) palliative CMT.

**Table 2 -:** Clinicopathological characteristics and outcomes of all ERa-positive patients.

Case	Age (years)	Sex	ASA	IHC score ERa	Lauren type	Tumor size (cm)	LN+/LN total	Lymphatic invasion	Venous invasion	Perineural invasion	pTNM	Final stage	DFS (months)	OS (months)	Site of recurrence	Status
1	63	Male	II	2	Diffuse	11.5	6+/47	Detected	Not detected	Detected	T1 N2 M0	IIA	115.9	115.9	-	Alive
2	78	Male	II	3	Diffuse	4.5	2+/40	Detected	Not detected	Detected	T4a N1 M0	IIIA	8.3	10.6	Peritoneum	Dead
3	34	Male	II	2	Diffuse	5.5	12+/45	Detected	detected	Detected	T4a N3a M0	IIIB	15.0	26.3	Peritoneum	Dead
4	68	Male	II	2	Diffuse	7.9	8+/50	Detected	Not detected	Detected	T4a N3a M0	IIIB	6.9	15.2	Peritoneum	Dead
5	62	Male	II	3	Diffuse	5.6	3/23	Not detected	Not detected	Detected	T4a N2 M0	IIIA	31.0	31.0	-	Alive
6	67	Male	II	2	Diffuse	12	5+/15	detected	Not detected	Detected	T3 N2 M0	IIIA	81.1	81.1	-	Alive

ASA, American Society of Anesthesiologists Classification; IHC, Immunohistochemistry; LN, lymph node; DFS, disease-free survival; OS, overall survival.

The median follow-up was 45.1 months, and the OS rate for the entire population was 53.1%. Among those six ERa-positive patients, three had disease recurrence and died. The site of recurrence in these three ERa-positive patients was peritoneal.

**Figure 2 -:**
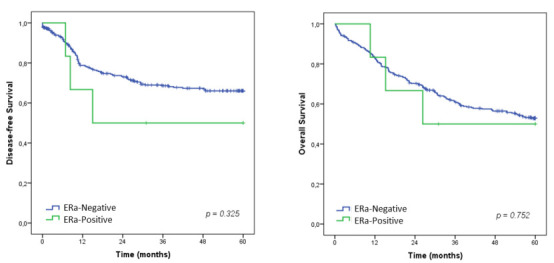
Disease-free survival and OS according to the ERa groups.

There was no difference in the OS rates between ERa-negative and ERa-positive groups (p=0.752). The median OS for ERa-positive patients was 26.3 months. Regarding DFS, no difference in survival was found between the groups (p=0.325). The median DFS for ERa-positive patients was 15 months (Figure 2).

## DISCUSSION

GC is a heterogeneous disease, and during diagnosis, it is mostly in an advanced stage. The treatment of GC depends on factors such as biomarkers, TNM staging, and the patient’s condition. In addition, despite advances in therapies, the prognosis of advanced GC patients remains poor^4^ ^,^ ^11^ ^,^ ^21^. Accordingly, the identification of tumor markers that can be used for diagnosis, predicting prognosis and response to therapy, seems promising^18^ ^,^ ^19^ ^,^ ^22^.

Thus, in the present study, we described a case series of ERa-positive gastric adenocarcinoma patients who underwent surgical resection and compared them with the negative ones. Although the frequency of positivity was low, we found homogeneity in relation to the characteristics of the patients. All GCs that exhibited ERa-positive had poorly differentiated histology, diffuse type, and lymph node metastasis, in agreement with that reported in the literature^23^ ^,^ ^25^. In fact, compared with other therapeutic targets, few studies have examined the expression of ERa in GC, so that there is still considerable controversy as to the expression level of ERa and its prognostic value in GC.

The first therapeutic target identified for GC treatment was HER-2. HER-2, also called ERB-2, is a tyrosine kinase receptor that, when mutated, has an effect on oncogenesis.^3^. It alters cell proliferation, cell differentiation, and program death and cell mobility. In addition, it is correlated with the progressive and metastatic potential of the tumor^15^. After the results of the ToGA trial, trastuzumab (anti-HER2 antibody) was approved for use in patients with positive expression for HER-2^3^ ^,^ ^15^.

In contrast, ER is a class of steroids that is involved in several functions of the body. In addition to regulating the development and growth of the human reproductive system, it also plays a role in the physiology of the cardiovascular, skeletal, and neuroendocrine systems^24^ ^,^ ^25^. Through its receptors, ERa and ERß, estrogen is able to translate signals into transcriptional responses. It is worth noting that both receptors, despite similar structures, have different functions^9^ ^,^ ^25^.

Estrogen plays a role through genomic and nongenomic pathways. In the genomic pathway, when estrogen is bound to its receptor, it is translocated into the nucleus, in which elements bind to the genomic DNA, regulating an expression of genes^19^. While in the nongenomic pathway, ERs interact with other signaling molecules, such as PI3K/Akt, or mitogen-activated protein kinase. ERs, namely, ERa and ERß, act in both pathways^5^ ^,^ ^13^.

Hormone receptors are extremely important in the role of oncogenesis in hormone-dependent tumors. In breast cancer, for example, ERa promotes tumorigenesis and progression of the tumor, while ERß expression is generally associated with inhibition of invasion, proliferation, and programmed cell death. In GC, however, the prognostic role of ER still remains controversial^.^ ^9^ ^,^ ^24^ ^,^ ^27^.

Tokunaga et al.^24^ were the first authors to study the correlation between hormone receptors and GC. In their study, the presence of estrogen and progesterone receptors was reported in 20% of gastric tumors. In addition, the results suggested that the GC could be influenced by hormonal factors^24^.

Regarding the frequency of expression reported in the literature, it can be noted that the rate of ERa expression in GC quite varies. Xu et al.^27^ observed an ERa positivity of 22.7% in patients with GC. However, the high frequency can be attributed to the predominance of tumors with undifferentiated histology among the evaluated patients (~80% of cases). In addition, the authors considered the weak cytoplasmic expression as positive^27^. In turn, Tang et al.^23^ observed the positive expression of ERa in 6% of the samples (9/150)^23^. Remarkably, there are studies in which no expression of ERa was found in GC, and other studies in which tumor exhibited only low level of expression^12^ ^,^ ^23^ ^,^ ^27^. In our study, similar to some previous studies, the frequency of ERa expression in GC patients was <2%. This can be explained in part by the high frequency of intestinal tumors in our cohort.

In the present series, we evaluate only the expression of ERa. In fact, some studies reported that only ERß exhibits a significant frequency of expression in GC, in contrast to others which mention that both receptors are expressed^23^ ^,^ ^25^. Furthermore, the prognostic impact of each ER subtype is also controversial, with different results regarding the presence of distant and lymph node metastasis, and in relation to OS^23^ ^,^ ^25^.

Tang et al.^23^ evaluated the expression of ERa, ERß, and androgen receptor by IHC in patients with GC and found that ERa-positive GC had a worse prognosis. In addition, its expression was associated with cell proliferation, migration, and invasion^13^. In our study, although the relationship with survival has not been statistically significant, patients with ERa-positive also presented pathological characteristics related to a worse prognosis, such as lymph node metastasis and diffuse Lauren histological type. Furthermore, among the three ER-positive patients who presented recurrence in our study, all had peritoneum metastasis, which refers to a worse prognosis - in addition to be related to the induction of epithelial-mesenchymal transition (EMT) phenotype^17^ ^,^ ^26^. EMT phenotype is related to invasion and metastasis of epithelial-derived cancers^26^, and the relationship between ERa expression and EMT has been previously reported^27^.

Presently, inconsistent associations of ERs with GC have been reported. Results from a meta-analysis showed that the rate of positivity for ERß expression in GC was higher than ERa, with different patterns in subtypes of tumors. GC positive for ERa was associated with poorly differentiated adenocarcinoma and worse OS. In contrast, the ERß positivity could have a protective effect against the invasiveness^25^. Another meta-analysis that included 11 studies also revealed an association between the expression of ERa with undifferentiated histology and worse OS; on the contrary, the expression of ERß was related to well-differentiated tumors and better survival^28^.

In the present study, from a cohort of 345 patients with GC, 6 (1.8%) were classified as ERa-positive. Positivity for ERa was associated with tumor size, diffuse/mixed Lauren histological type, presence of perineural invasion, and lymph node metastasis. Despite presenting the characteristics associated with a worse prognosis and advanced disease, there was no significant difference in survival outcomes. However, this can be attributed to the low number of patients positive for ERa in the present series.

There were some limitations in the present study, inherent in retrospective studies, like selection bias, which could interfere in results considering the interaction between variables. Only patients undergoing surgical resection were included. Thus, we do not know whether the expression of ER has differences in patients undergoing palliative treatment. Still, variations in results compared with other studies are predicted due to different evaluation criteria for IHC results and antibody clones used^8^ ^,^ ^23^ ^,^ ^28^. Still, the analysis only considered ERa, the subtype which is more associated with GC prognosis in literature^9^ ^,^ ^28^. Variations can also be attributed with respect to tumor sampling. In this study, patients were assessed through the TMA construction. This may increase the chance of false-negative results in the case of markers where the expression is restricted. However, we followed the guidelines and, as suggested, we used three tissue cores of tumor from each patient which are recommended for an adequate assessment of tumor heterogeneity^10^.

## CONCLUSIONS

The expression of ERa in GC was associated with diffuse histology and presence of lymph node metastasis and may suggest a role as a marker related to tumor progression and worse prognosis. Also, a high rate of peritoneal recurrence was observed in ERa-positive patients. Since the frequency of ERa expression seems to be low, studies that involve larger cohorts of patients and standardization in the methods of IHC evaluation are requested to define its impact on patient survival in GC.

## References

[1] Ajani JA, In H, Sano T (2017). American Joint Committee on Cancer (AJCC). Cancer Staging
Manual.

[2] Allred DC, Bustamante MA, Daniel CO, Gaskill HV, Cruz AB (1990). Immunocytochemical analysis of estrogen receptors in human breast
carcinomas. Evaluation of 130 cases and review of the literature regarding
concordance with biochemical assay and clinical relevance. Arch Surg.

[3] Bang YJ, Van Cutsem E, Feyereislova A, Chung HC, Shen L, Sawaki A, Lordick F, Ohtsu A, Omuro Y, Satoh T, Aprile G, Kulikov E, Hill J, Lehle M, Rüschoff J, Kang YK, ToGA Trial Investigators (2010). Trastuzumab in combination with chemotherapy versus chemotherapy
alone for treatment of HER2-positive advanced gastric or gastro-oesophageal
junction cancer (ToGA): a phase 3, open-label, randomised controlled
trial. Lancet.

[4] archi LC, Ramos MFKP, Dias AR, Andreollo NA, Weston AC, Lourenço LG, Malheiros CA, Kassab P, Zilberstein B, Ferraz ÁAB, Charruf AZ, Brandalise A (2020). II Brazilian consensus on gastric cancer by the Brazilian Gastric
Cancer Association. Arq Bras Cir Dig.

[5] Barzi A, Lenz AM, Labonte MJ, Lenz HJ (2013). Molecular pathways: Estrogen pathway in colorectal
cancer. Clin Cancer Res.

[6] Bray F, Ferlay J, Soerjomataram I, Siegel RL, Torre LA, Jemal A (2018). Global cancer statistics 2018: GLOBOCAN estimates of incidence
and mortality worldwide for 36 cancers in 185 countries. CA Cancer J Clin.

[7] Fontana E, Smyth EC (2016). Novel targets in the treatment of advanced gastric cancer: a
perspective review. Ther Adv Med Oncol.

[8] Gan L, He J, Zhang X, Zhang YJ, Yu GZ, Chen Y, Pan J, Wang JJ, Wang X (2012). Expression profile and prognostic role of sex hormone receptors
in gastric cancer. BMC Cancer.

[9] Ge H, Yan Y, Tian F, Wu D, Huang Y (2018). Prognostic value of estrogen receptor α and estrogen receptor β
in gastric cancer based on a meta-analysis and The Cancer Genome Atlas
(TCGA) datasets. Int J Surg.

[10] Ilyas M, Grabsch H, Ellis IO, Womack C, Brown R, Berney D, Fennell D, Salto-Tellez M, Jenkins M, Landberg G (2013). Guidelines and considerations for conducting experiments using
tissue microarrays. Histopathology.

[11] Japanese Gastric Cancer Association (2017). Japanese gastric cancer treatment guidelines 2014 (ver.
4). Gastric Cancer.

[12] Kojima O, Takahashi T, Kawakami S, Uehara Y, Matsui M (1991). Localization of estrogen receptors in gastric cancer using
immunohistochemical staining of monoclonal antibody. Cancer.

[13] Kousteni S, Bellido T, Plotkin LI, O’Brien CA, Bodenner DL, Han L, Han K, DiGregorio GB, Katzenellenbogen JA, Katzenellenbogen BS (2001). Nongenotropic, sex-nonspecific signaling through the estrogen or
androgen receptors: dissociation from transcriptional
activity. Cell.

[14] Lee SY, Oh SC (2016). Changing strategies for target therapy in gastric
cancer. World J Gastroenterol.

[15] Lei YY, Huang JY, Zhao QR, Jiang N, Xu HM, Wang ZN, Li HQ, Zhang SB, Sun Z (2017). The clinicopathological parameters and prognostic significance of
HER2 expression in gastric cancer patients: a meta-analysis of
literature. World J Surg Oncol.

[16] Matboli M, El-Nakeep S, Hossam N, Habieb A, Azazy AE, Ebrahim AE, Nagy Z, Abdel-Rahman O (2016). Exploring the role of molecular biomarkers as a potential weapon
against gastric cancer: A review of the literature. World J Gastroenterol.

[17] Nishii T, Yashiro M, Shinto O, Sawada T, Ohira M, Hirakawa K (2009). Cancer stem cell-like SP cells have a high adhesion ability to
the peritoneum in gastric carcinoma. Cancer Sci.

[18] Pereira MA, Ramos MFKP, Dias AR, Faraj SF, Ribeiro RRE, de Castria TB, Zilberstein B, Alves VAF, Ribeiro U, de Mello ES (2019). Expression Profile of Markers for Targeted Therapy in Gastric
Cancer Patients: HER-2, Microsatellite Instability and PD-L1. Mol Diagn Ther.

[19] Ramos MFKP, Pereira MA, Amorim LC, de Mello ES, Faraj SF, Ribeiro U, Hoff PMG, Cecconello I, de Castria TB (2020). Gastric cancer molecular classification and adjuvant therapy: Is
there a different benefit according to the subtype?. J Surg Oncol.

[20] Ramos MFKP, Pereira MA, Yagi OK, Dias AR, Charruf AZ, Oliveira RJ, Zaidan EP, Zilberstein B, Ribeiro-Júnior U, Cecconello I (2018). Surgical treatment of gastric cancer: a 10-year experience in a
high-volume University Hospital. Clinics (Sao Paulo).

[21] Smyth EC, Verheij M, Allum W, Cunningham D, Cervantes A, Arnold D, ESMO Guidelines Committee (2016). Gastric cancer: ESMO Clinical Practice Guidelines for diagnosis,
treatment and follow-up. Ann Oncol.

[22] Sohn BH, Hwang JE, Jang HJ, Lee HS, Oh SC, Shim JJ, Lee KW, Kim EH, Yim SY, Lee SH (2017). Clinical Significance of Four Molecular Subtypes of Gastric
Cancer Identified by The Cancer Genome Atlas Project. Clin Cancer Res.

[23] Tang W, Liu R, Yan Y, Pan X, Wang M, Han X, Ren H, Zhang Z (2017). Expression of estrogen receptors and androgen receptor and their
clinical significance in gastric cancer. Oncotarget.

[24] Tokunaga A, Kojima N, Andoh T, Matsukura N, Yoshiyasu M, Tanaka N, Ohkawa K, Shirota A, Asano G, Hayashi K (1983). Hormone receptors in gastric cancer. Eur J Cancer Clin Oncol.

[25] Ur Rahman MS, Cao J (2016). Estrogen receptors in gastric cancer: Advances and
perspectives. World J Gastroenterol.

[26] Xia P, Xu XY (2017). Epithelial-mesenchymal transition and gastric cancer stem
cell. Tumour Biol.

[27] Xu CY, Guo JL, Jiang ZN, Xie SD, Shen JG, Shen JY, Wang LB (2010). Prognostic role of estrogen receptor alpha and estrogen receptor
beta in gastric cancer. Ann Surg Oncol.

[28] Zhang D, Ku J, Yi Y, Zhang J, Liu R, Tang N (2019). The prognostic values of estrogen receptor alpha and beta in
patients with gastroesophageal cancer: A meta-analysis. Medicine (Baltimore).

